# Rhinoscleroma presenting as a nasal-palatal mass with airway obstruction

**DOI:** 10.12688/f1000research.2-124.v1

**Published:** 2013-05-09

**Authors:** Mark C Domanski, Alexander Rivero, David E Kardon

**Affiliations:** 1 Division of Otolaryngology, Washington Adventist Hospital, Takoma Park, Maryland, 20912, USA; 2 Division of Otolaryngology, George Washington University, Washington D.C., 20037, USA; 3 Department of Pathology, Washington Adventist Hospital, Takoma Park, Maryland, 20912, USA

## Abstract

We report a case of a 45-year-old male with severe rhinoscleroma. The patient presented to the emergency room with dyspnea from a long-standing nasal-palatal mass. A tracheostomy was required for airway control. While dyspnea in the presence of an upper airway mass is typical of malignancy, consideration of non-oncological etiologies is important. We review the epidemiology, pathology, diagnosis, and treatment of rhinoscleroma.

## Introduction

Rhinoscleroma is a chronic bacterial infection caused by *Klebsiella rhinoscleromatis*, a Gram-negative, non-motile, encapsulated bacillus. Due to the low infectivity of the bacteria, chronic exposure is required in order to establish infection. Rhinoscleroma is more frequent in the developing world, and is likely a secondary complication as a result of underdeveloped hygiene infrastructures, poor access to antibiotics, and overcrowded living conditions. Most cases are found in Central America, Africa and the Middle East ^1^. The prevalence of sporadic cases outside of endemic areas is usually attributed to immigration ^2^. Though rhinoscleroma can involve any structure of the upper respiratory tract, *Klebsiella rhinoscleromatis* has an affinity for nasal mucosa and thus is present in the nasal cavity in 95–100% of cases ^3^. It can also be found in the nasopharynx (18–43%), larynx (15–40%), trachea (12%), and bronchi (2–7%) ^4^. Here, we present a case with both nasal and palatal involvement resulting in airway obstruction.

## Case report

A 45-year-old Central American male presented with a 13-year palatal mass, and new onset stridor in the background of chronic dyspnea. He denied weight loss and night sweats. He worked as a day laborer, drank socially, but never smoked. He had been unable to breathe out of his nose for at least thirty years.

**Figure 1.**
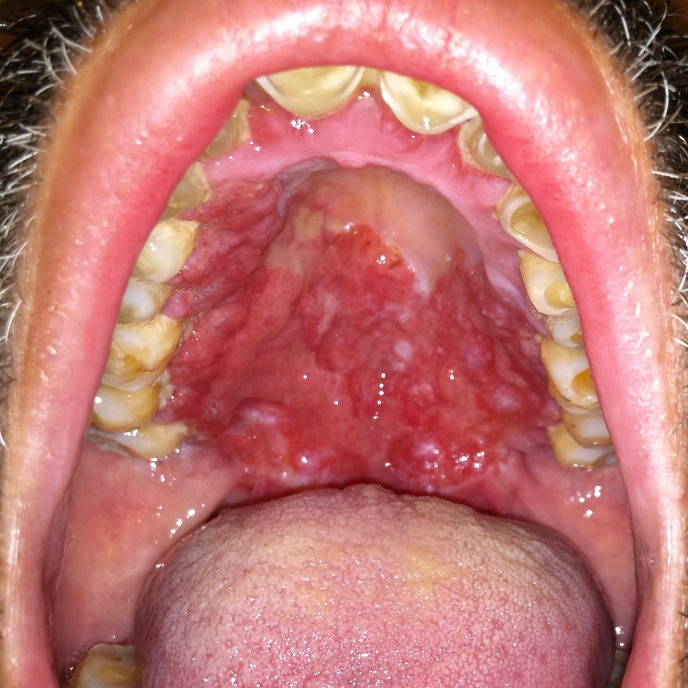
Oral cavity showing a plaque-like erythematous mass involving the gingiva, hard and soft palates.

Nasal endoscopy showed obstructed choana bilaterally. Inspection of the oral cavity showed a hard, plaque like growth involving the hard and soft palates, pharynx, and marked foreshortening of the palatoglossal folds ( Figure 1). Dentition was poor. Endoscopic visualization of the larynx could only be performed transorally. The patient’s airway was tight at the level of the palatoglossal folds and base of the tongue. The vocal cords and epiglottis were uninvolved.

**Figure 2.**
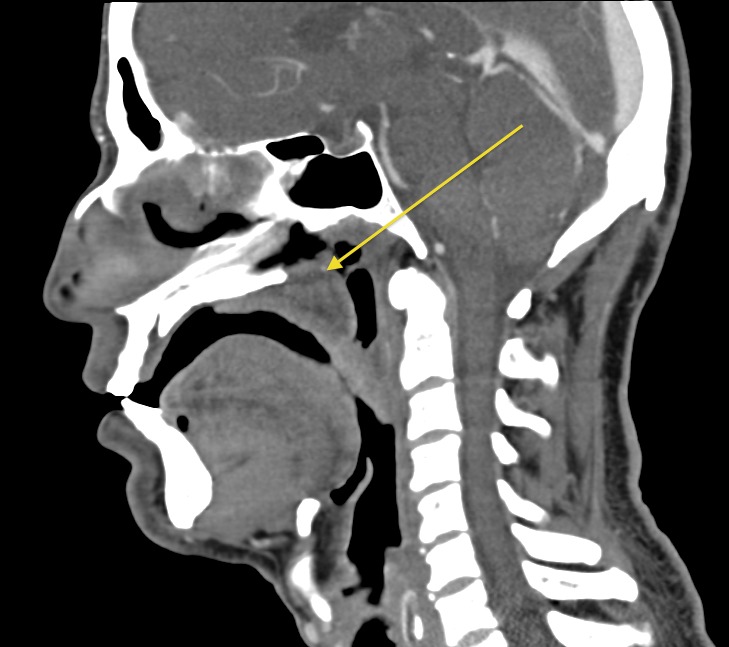
CT neck with contrast in sagittal plane. Heterogeneous soft tissue is present in the nasopharynx.

A computed tomography (CT) scan confirmed a palatal mass, and obstructed choana. Thickening of the uvula, and hard and soft palate mucosa was noted. No palatal bony obstruction or lymphadenopathy was seen ( Figure 2).

**Figure 3.**
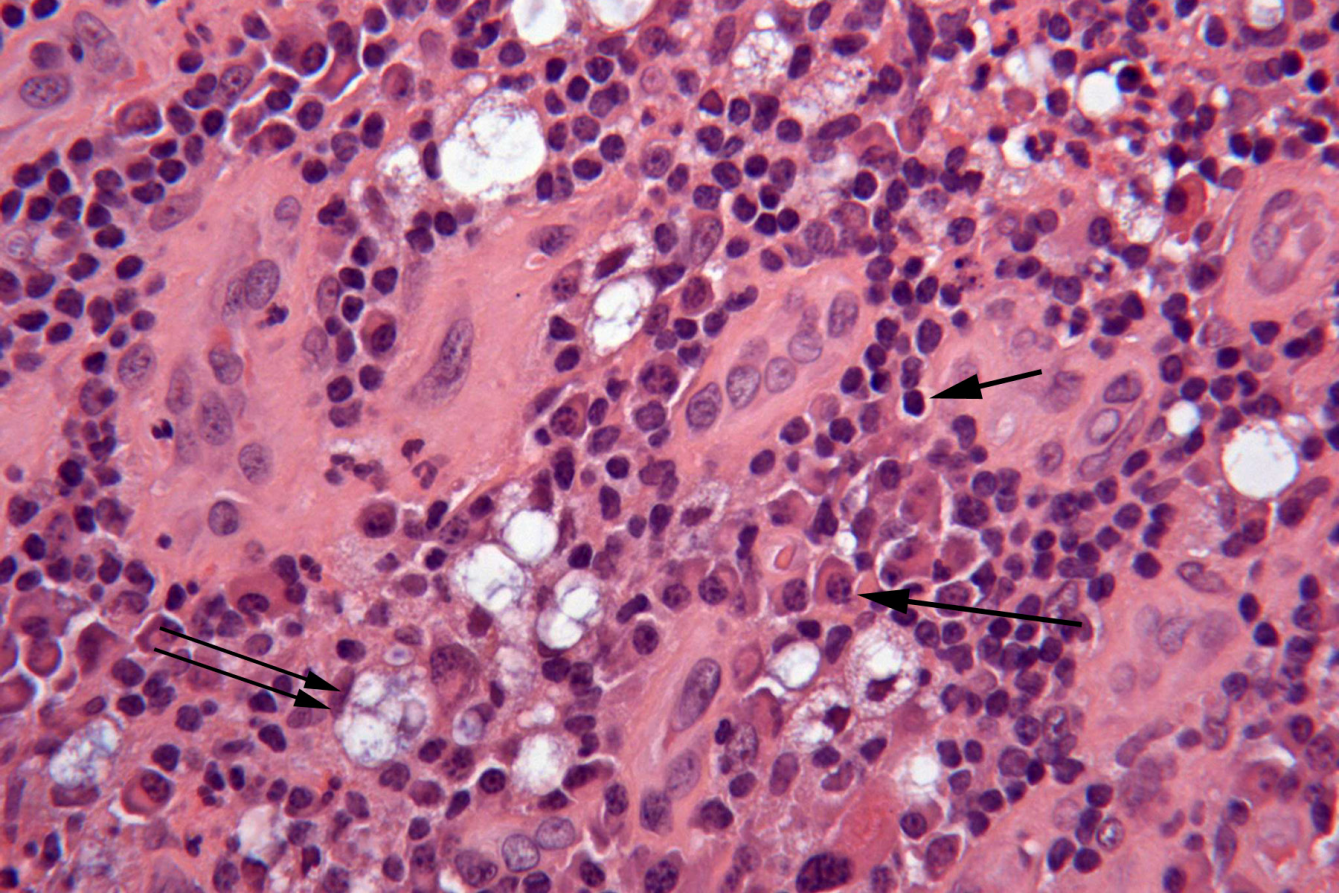
H&E stain (400×) demonstrated a mixture of plasma cells (arrow), lymphocytes (short arrow) and vacuolated macrophages (Mikulicz cells) (double arrow).

**Figure 4.**
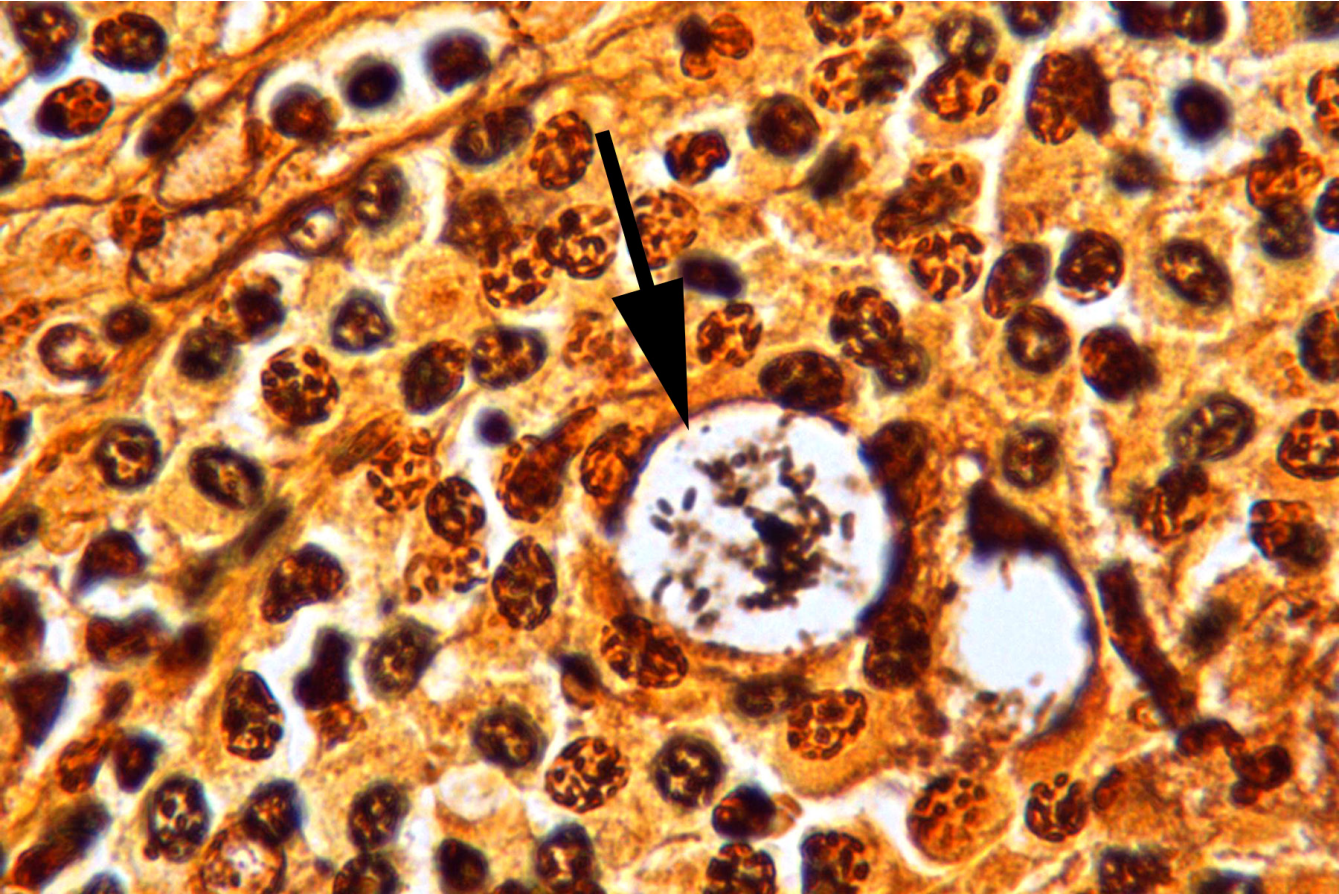
Steiner stain, (1000×) with rod-shaped bacilli within a vacuolated macrophage (Mikulicz cell) (arrow).

A local awake tracheostomy was performed to provide a secure airway. A palatal biopsy was sent for analysis and demonstrated squamous mucosa with a dense, mixed inflammatory infiltrate containing abundant plasma cells and scattered vacuolated macrophages (Mikulicz cells) ( Figure 3). A Warthin-Starry stain revealed rod-shaped bacilli within the vacuolated macrophages. The bacilli were morphologically consistent with *Klebsiella* ( Figure 4).

The patient was treated with ciprofloxacin 500 mg BID for 12 weeks. His airway symptoms improved and he was later decannulated without sequelae. He declined surgical nasal airway debridement.

## Discussion

Rhinoscleroma generally progresses in three stages. The initial stage is the catarrhal or exudative phase. This is followed by the proliferative or granulomatous phase, which finally evolves into the cicatricial phase ^2^. During the catarrhal stage, patients may have persistent rhinitis and mucopurulent discharge. In the second stage, inflamed mucosa coalesces to form granulomas. These granulomas may infiltrate other portions of the airway and then scar, giving rise to the third or cicatricial stage ^2^. These stages usually do not exist independently. In many cases of rhinoscleroma, the presence of all three stages can be found at the time of diagnosis.

Rhinoscleroma is spread by person-to-person transmission. However due to the low infectivity of the pathogen, transmission requires a chronic exposure. It has also been proposed that an altered immune response along with an alteration in the CD4+ and CD8+ proportion leads to ineffective macrophage production that are susceptible to bacterial replication ^5^.

A high degree of suspicion is warranted when patients present with persistent, unremitting rhinitis or nasal obstruction unexplained by other causes. The differential diagnosis of such symptoms should include rhinoscleroma, as well as tuberculosis, syphilis, Wegener’s granulomatosis, lymphomas as well as more common carcinomas. Histopathologic evidence of rhinoscleroma includes granulomatous inflammation with large vacuolated histiocytes known as Mikulicz cells ^6^. Canalis *et al.* proposed that these Mikulicz cells arise from histiocytes that migrate to areas where neutrophils have failed to contain the *Klebsiella* infection ^7^. The histiocytes, however, are unable to lyse their phagocytosed *Klebsiella* cells, leading to the dilation of their vacuoles ^7^. Positive culture of rhinoscleroma on MacConkey agar is diagnostic, though culture is only positive in 50–60% of patients. Thus, it is key to have high clinical suspicion in conjunction with positive histopathologic evidence to confirm the diagnosis.

Historically, treatment of rhinoscleroma was with tetracyclines and aminoglycosides such as streptomycin. However, a prospective study done in the Mayo Clinic, USA, by Andraca *et al.* in 1993 demonstrated the efficacy of fluoroquinolones ^8^. Treatment with fluoroquinolones also confers the benefit of a lower side-effect profile. Dosing of the antibiotic is variable between different studies, but most agree that long-term therapy for months and sometimes years is necessary to adequately treat the infection ^3, 7, 8^. Despite treatment, recurrence has been reported in up to 25% of cases at 10 years ^2, 4^. Consideration should be made when addressing whether a patient requires surgical de-bulking of the scar in rhinoscleroma formed during the cicatricial stage. Indications for surgical de-bulking include airway patency, treatment of bulky disease, and cosmesis.

## Conclusion

Rhinoscleroma is due to chronic and indolent *Klebsiella* infection. Symptoms may include chronic, unremitting rhinitis or nasal obstruction that is present for years. The presenting symptom can also be more dramatic, such as airway compromise, as seen in this case. A diagnosis of rhinoscleroma is made via pathological specimens. Communication between the clinician and the pathologist as to the possibility of non-oncological processes can aid in determining the diagnosis. A Warthin-Starry stain demonstrating rod-shaped bacilli within vacuolated macrophages (Mikulicz cells) is classic for rhinoscleroma. Mainstay of treatment is long-term fluoroquinolones. Evaluation of airway patency is critical and surgical intervention may be required.

## Consent

Written informed consent for publication of clinical details and clinical images was obtained from the patient.
