# Supplementary material for: Rhinoscleroma presenting as a nasal-palatal mass with airway obstruction
Source: F1000Res. 2013 May 9;2:124. [Version 1] doi: 10.12688/f1000research.2-124.v1 (PMC3814917; doi:10.12688/f1000research.2-124.v1)
Supplement: Peer review history

# Peer review: Referee response for version 1

**Published:** 2013-05-31

**Authors:** Alkis Psaltis

**Affiliations:** 1 Stanford University Medical Center, Stanford, CA, USA

The authors present an interesting case report of a condition not commonly seen in developed countries. Its unusual presentation reaffirms the need for otolaryngologists to consider infectious processes in the work up of sinonasal and nasopharyngeal masses. The paper is concise, well written and easy to follow. It provides a nice summary of rhinoscleroma particularly of the histopathophysiological features essential for diagnosis.

Additional photographs demonstrating the encroachment of the mass on the pharyngeal airway (i.e. with the use of a tongue depressor), a nasal endoscopic view and a post treatment view may have been helpful in providing more visual information for the reader.

I agree with the previous reviewer’s concern regarding the need for an awake tracheotomy for the management of the airway. Given the superior appearance of the mass on the sagittal CT scan and  the fact that the authors state that an adequate visualization of the uninvolved vocal cords and epiglottis was obtained with transoral endoscopy, why did the authors not consider awake fiber-optic endoscopic intubation or at least mention this as an alternative for managing the airway. 

The discussion of the response of the mass to treatment is a little brief. I think additional information would be useful to the reader such as: Percentage decrease in the size of the mass with treatment, how the authors deemed that 12 weeks of treatment was appropriate, progress post treatment and also any scheduled future follow up.

I have read this submission. I believe that I have an appropriate level of expertise to confirm that it is of an acceptable scientific standard, however I have significant reservations, as outlined above.

# Peer review: Referee response for version 1

**Published:** 2013-05-16

**Authors:** Allen M Seiden

**Affiliations:** 1 Department of Otolaryngology, College of Medicine, University of Cincinnati, Cincinnati, OH, USA

This is an interesting case report describing an unusual presentation for rhinoscleroma, an infectious problem that we see rarely in the USA. It is well written, and well-organized. It would have been interesting to include patient photos post treatment. It can be a difficult diagnosis to make, so a more thorough literature review discussing potential physical findings on presentation would also have been helpful.

I have read this submission. I believe that I have an appropriate level of expertise to confirm that it is of an acceptable scientific standard.

# Peer review: Referee response for version 1

**Published:** 2013-05-15

**Authors:** Rakesh K Chandra

**Affiliations:** 1 Department of Otolaryngology - Head and Neck Surgery, Northwestern University Feinberg School of Medicine, Chicago, IL, USA

The manuscript is an excellent review of the topic and is well illustrated with a clinical photo, radiology, and histopathology. My only area of concern is that I wonder whether it was indeed necessary to perform a tracheotomy on this patient. If the glottis was visible transorally, and given the findings on the sagittal radiologic image shown, it looks like the patient could have been intubated. Also the lesion is too superior to cause true stridor. It could be argued that tracheotomy was advised because the patient would have had difficulty managing secretions and there was concern the lesion would swell or bleed upon biopsy, but it is difficult to imagine true stridor and that this patient couldn’t have been intubated. The report would be improved if the authors justify their decision to perform the tracheotomy in the light of these comments

Title and Abstract are appropriate and the conclusions are otherwise balanced and justified.

I have read this submission. I believe that I have an appropriate level of expertise to confirm that it is of an acceptable scientific standard, however I have significant reservations, as outlined above.
